# Fuzzy inference system enabled neural network feedforward compensation for position leap control of DC servo motor

**DOI:** 10.1038/s41598-024-71647-1

**Published:** 2024-09-06

**Authors:** Zhiwen Huang, Yuting Yan, Yidan Zhu, Jiajie Shao, Jianmin Zhu, Dianjun Fang

**Affiliations:** 1 School of Mechanical Engineering, University of Shanghai for Science and Technology, Shanghai, China; 2 College of Design and Engineering, National University of Singapore, Singapore, Singapore; 3 School of Mechanical Engineering, Tongji University, Shanghai, China

**Keywords:** Fuzzy inference system, Feedforward compensation control, Artificial neural network, Position leap control, DC servo motor, Electrical and electronic engineering, Mechanical engineering

## Abstract

To improve dynamic performance and steady-state accuracy of position leap control of the direct current (DC) servo motor, a fuzzy inference system (FIS) enabled artificial neural network (ANN) feedforward compensation control method is proposed in this study. In the method, a proportional-integral-derivative (PID) controller is used to generate the baseline control law. Then, an ANN identifier is constructed to online learn the reverse model of the DC servo motor system. Meanwhile, the learned parameters are passed in real-time to an ANN compensator to provide feedforward compensation control law accurately. Next, according to system tracking error and network modeling error, an FIS decider consisting of an FI basic module and an FI finetuning module is developed to adjust the compensation quantity and prevent uncertain disturbance from undertrained ANN adaptively. Finally, the feasibility and efficiency of the proposed method are verified by the tracking experiments of step and square signals on the DC servo motor testbed. Experimental results show that the proposed FIS-enabled ANN feedforward compensation control method achieves lower overshoot, faster adjustment, and higher precision than other comparative control methods.

## Introduction

Since it has the characteristics of high efficiency, rapid response, and reliable performance, electric motors have been widely used in many fields, such as aerospace^1,2^, electric automobile^3,4^, numerical control equipment^5,6^. In particular, benefiting from the advantages of small size, economical price, and high accuracy^7^, the direct current (DC) servo motors have been the main actuators of subsystems, such as electronic throttle valves^8^, joystick system actuator^9^, and vehicle electric power steering^10^. Nevertheless, the development of controllers for DC motor servo systems confronts a multitude of challenges stemming from their inherent nonlinearities and uncertainties^10^, which complicate the design process and require innovative strategies to ensure precise and robust performance.

Over the years, many efforts have been made to improve the position control precision and performance of the DC servo motors. Correspondingly, various control methods have been developed by researchers, such as intelligent H-infinity control^11^, sliding mode control^12,13^, model predictive control^14^, adaptive control^15^, nonlinear robust control^16^, etc. Although these methods have enhanced the motion control precision of DC servo motors in different aspects, their performance heavily depends on accurate system modeling based on rich prior knowledge^17^. It brings a big challenge in the industrial application of these advanced control methods due to the nonlinear and time-varying characteristics of the control systems.

In practice, the proportional-integral-derivative (PID) method has been used for position-tracking control of DC servo motors due to its merits of algorithm simplicity, good usability, and strong anti-interference^18^. It is difficult to achieve the simultaneous optimum of dynamic performance and steady-state accuracy by only tuning the PID parameters due to the time-varying characteristics of the servo motor system^19^. Generally, it is combined with the advanced feedforward compensation method to enhance tracking performance in the position loop of the DC servo motor. Zhou et al. fused backstepping adaptive control and feedforward gap compensation and improved the tracking accuracy and stability of the DC servo motor system while suppressing adverse effects of transmission clearance^20^. Lin et al. proposed an ideal cascade integral system with feedforward compensation for improving the position-tracking performance of the DC servo motor system^21^. Yao et al. integrated adaptive control and extended state observer via a feedforward cancellation technique, obtaining the high-accuracy control of the DC servo motor system^22^. Particularly, the high tracking performance of these feedforward strategies still relies on the accurate mathematical model of the DC servo motor system. Its complex nonlinearity and time variability still significantly limit the practical application of these compensation methods in the DC servo motor system.

Artificial neural networks (ANN) can accurately approximate nonlinear systems due to their strong self-learning capability^23–26^, thereby improving position control performance based on their adaptive modeling of the control systems. For instance, Wang et al. presented an ANN-driven nonlinear motor control method and enhanced the position-tracking performance of the servo motor system^27^. Yang et al. designed an ANN-based adaptive tracking controller and guaranteed the transient performance of a hydraulic servo manipulator system^28^. Chuei and Cao proposed a repetitive controller based on an extreme learning machine with a single-layer feedforward network, improving the tracking accuracy of a brushless DC servo motor^29^. Yang et al. developed a feedback control scheme with an ANN-based unknown dynamics compensation for the DC motor system, achieving high-accuracy tracking performance^30^. Huang et al. proposed a hybrid control method based on ANN feedforward compensation, enhancing both the transient response and steady-state performance of the magnetic levitation position control^31^. Although these methods can improve the control performance by leveraging neural networks to identify the control system, its modeling accuracy heavily depends on training samples. When tracking step and square signals with leap characteristics, the training samples are significantly insufficient at the moment of signal jump, which makes it difficult to train ANN effectively. It inevitably leads to output uncertainty of neural networks, thereby causing control uncertainty of these control methods to some extent.

Recently, benefitting from the merits of clear interpretation, simple computation, and reliable reasoning, a fuzzy inference system (FIS) has been introduced into nonlinear control systems to address the control uncertainty^32–35^. Sun et al. applied fuzzy supervised learning to predict nonlinear actuator faults and model uncertainties with insufficient prior knowledge, achieving higher tracking precision and better control stability of the fault-tolerant spacecraft attitude control^36^. Khaniki et al. used an interval type-2 FIS to estimate the discontinuous sign function in the proposed adaptive nonsingular fast terminal sliding mode method, eliminating the uncertain chattering phenomenon in the conventional sliding mode controller^37^. Santoso et al. introduced a type-2 evolutionary FIS to a nonlinear system identification technique, and simulation results demonstrated that it can capture more uncertainties without complex mathematical equations^38^. Tang et al. utilized a fuzzy inference block to address the control uncertainty caused by the undertrained neural network, improving the transient performance of position control of the magnetic levitation system^39^. However, most of these FIS-based methods mainly focused on the control uncertainty in the simulation environment, which ignores the time-varying difference between the simulation model and the real system. Furthermore, to address the uncertainty caused by neural networks, the methods only consider the internal error and its change in the control system, while ignoring the internal state of the neural network, such as the change in the learning weights. This weakens the active suppression of control uncertainty caused by the undertrained neural network, thereby limiting the control performance of the time-varying system.

To solve the above problems, the FIS-enabled ANN feedforward compensation control method is proposed for position leap control of the DC servo motor. It consists of a PID controller, an ANN identifier, an ANN compensator, and an FIS decider that consists of an FI basic module and an FI finetuning module. The main innovations and contributions are highlighted in the following:

1. An FIS-enabled ANN feedforward compensation method is proposed to improve both dynamic performance and steady-state accuracy of the position leap control of the DC servo motor without building its accurate mathematical model.
2. An ANN identifier is designed to online learn the inverse model of the DC servo motor system while an RNN controller is designed to provide dynamic feedforward compensation for the position leap control of the DC servo motor in real-time.
3. An FIS decider consisting of an FI basic module and an FI finetuning module is designed to suppress uncertainty interference of the undertrained ANN identifier and accurately adjust the feedforward compensation control quantity of the ANN compensator.
4. Tracking experiments are carried out to demonstrate the effectiveness and advancement of the FIS-enabled ANN feedforward compensation method, results show that it can lower overshoot, reduce settling time, and improve steady-state precision.

The remainder of this research is organized in the following. Section "Proposed control method" describes the overall control structure and integrated control law in detail. Then, the FIS decider consisting of an FI basic module and an FI finetuning module is designed in Section "FIS decider design". Next, experimental platform and position tracking results are presented and analyzed in Section "Experimental verification". Hyperparameter sensitivity and anti-interference performance are discussed in Section "Discussion". Finally, Section "Conclusion" draws the conclusion of this research.

## Proposed control method

### Overall control structure

**Fig. 1:**
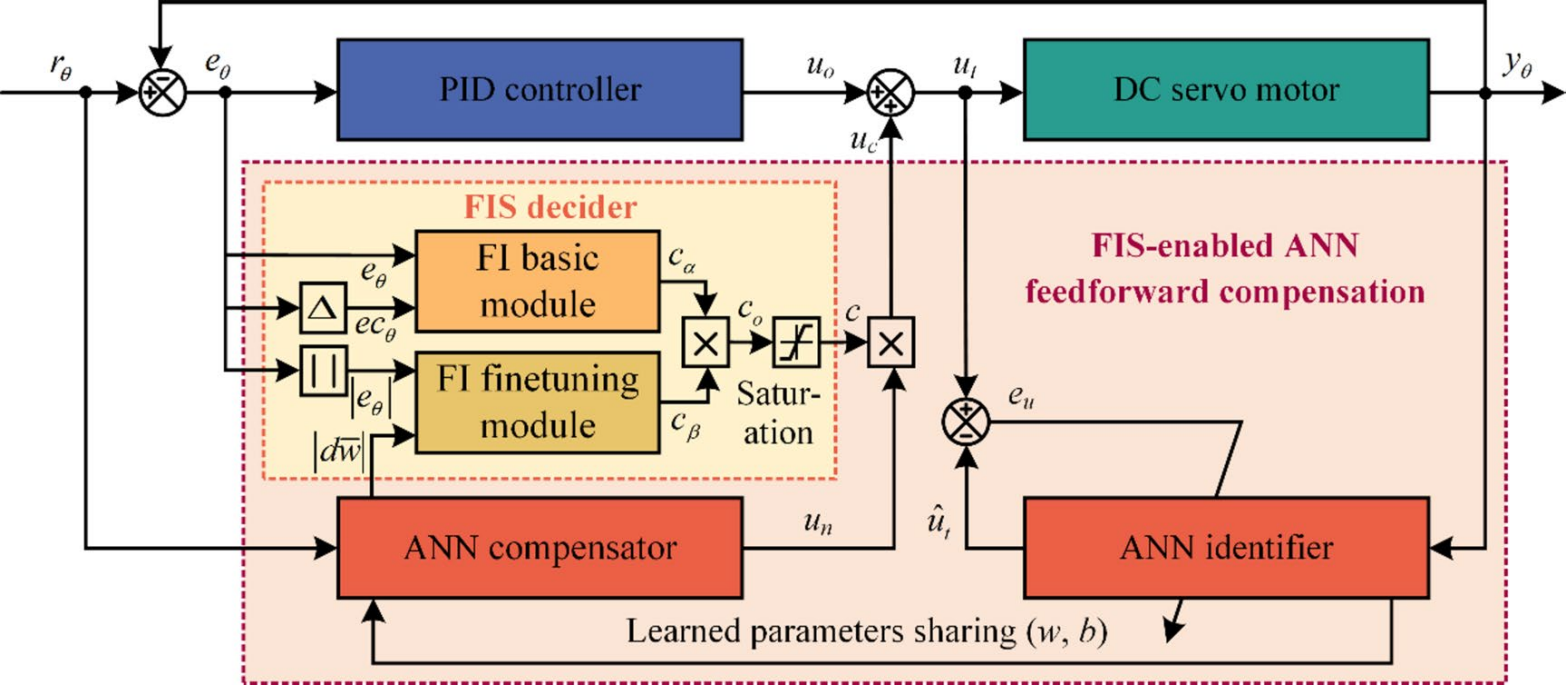
The overall structure of the FIS-enabled ANN feedforward compensation method.

In this study, the FIS-enabled neural network feedforward compensation method is proposed for position leap control of the DC servo motor. The overall control structure of the proposed method is illustrated in Fig. 1. It comprises four modules, that is, a PID controller, an ANN identifier, an ANN compensator, and an FIS decider that consists of an FI basic module and an FI finetuning module.

The PID controller with a clear principle and a simple algorithm is used as a baseline controller. Its main function is to maintain the control stability at the early control phase and produce learning samples for the ANN identifier. Furthermore, the ANN identifier is designed to online identify an inverse model of the DC servo motor system. Sharing the same network structure and learned parameters as the ANN identifier, the ANN compensator is constructed to provide accurate feedforward compensation for position control of the DC servo motor.

In particular, at the early control phase or the leap instantaneous of the tracking signal, the online samples are insufficient to train an effective ANN identifier. It can’t learn the inverse model of the DC servo motor system accurately, thereby resulting in control uncertainty of the ANN compensator. Therefore, the FIS decider including an FI basic module and an FI finetuning module is developed to adaptively adjust the controlled quantity of the ANN compensator. The FI basic module and the FI finetuning module are applied to the coarse tuning and the finetuning of compensation quantity, respectively. Ultimately, after the combined effect of these modules, the proposed FIS-enabled feedforward compensation method can improve control performance as well as reduce control uncertainty.

### Integrated control law

As shown in Fig. 1, the integrated control law of the proposed FIS-enabled ANN feedforward compensation method consists of the baseline control quantity ${u}_{o}$ and the compensated control quantity ${u}_{c}$. Therefore, the overall control law ${u}_{t}$ can be expressed as follows

1 $${u}_{t}={u}_{o}+{u}_{c}$$

The baseline control quantity ${u}_{o}$ is from the PID controller. Its expression is as follows

2 $${u}_{o}={k}_{p}{e}_{\theta }\left(k\right)+{k}_{i}\sum_{i=0}^{k}{e}_{\theta }\left(i\right)\Delta t+{k}_{d}\frac{{e}_{\theta }\left(k\right)-{e}_{\theta }\left(k-1\right)}{\Delta t}$$

where ${e}_{\theta }\left(k\right)$ is the control error at $k$ moment, ${e}_{\theta }\left(k-1\right)$ is the control error at $k-1$ moment, $\Delta t$ is the control sampling interval. Besides, ${k}_{p}$, ${k}_{i}$, and ${k}_{d}$ are the proportion, integration, and differentiation gains of the PID controller, respectively.

Additionally, as shown in Fig. 1, the compensated control quantity ${u}_{c}$ is equal to the reasoning output $c$ of the FIS decider multiplied by the control output ${u}_{n}$ of the ANN compensator. Its expression is as follows

3 $${u}_{c}=c\cdot {u}_{n}$$

**Fig. 2:**
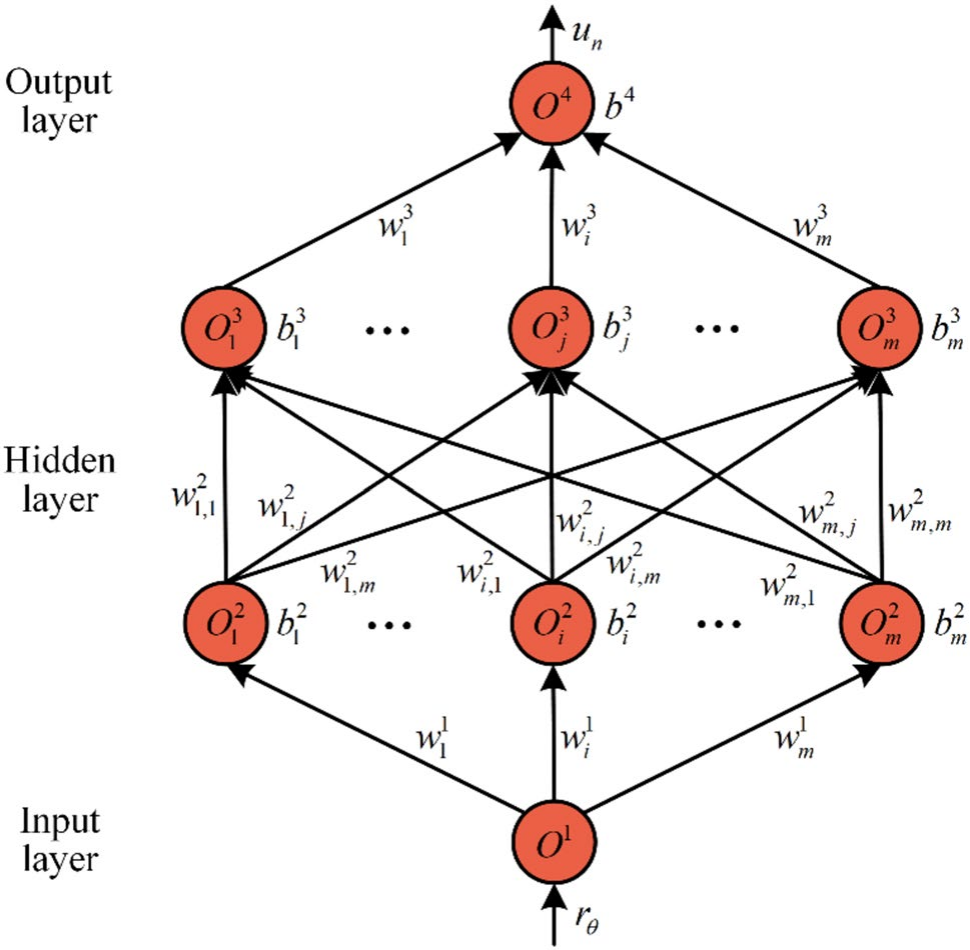
Network structure of the ANN compensator.

The control output ${u}_{n}$ is calculated by the forward propagation of the ANN compensator. As illustrated in Fig. 2, it consists of one input layer with one neuron, two hidden layers with five neurons, and one output layer with one neuron. Its calculation process is as follows

4 $${O}^{1}={r}_{\theta }$$

5 $${O}_{i}^{2}=\sigma \left({\omega }_{i}^{1}\cdot {O}^{1}+{b}_{i}^{2}\right)$$

6 $${O}_{j}^{3}=\sigma \left(\sum_{i=1}^{m}{{\omega }_{i,j}^{2}\cdot O}_{i}^{2}+{b}_{j}^{3}\right)$$

7 $${u}_{n}={O}^{4}=\sum_{j=1}^{m}{{\omega }_{i}^{3}\cdot O}_{j}^{3}+{b}^{4}$$

where ${O}^{1}$ is the output of the input layer, ${O}_{i}^{2}$ is the *i*-th output of the first hidden layer, ${O}_{j}^{3}$ is the *j*-th output of the second hidden layer, and ${O}^{4}$ is the output of the output layer. In addition, $\sigma (\cdot )$ represents the Sigmoid activation function, ${\omega }_{i}^{1}$ is a weight between the input layer and the *i*-th neuron of the first hidden layer, ${b}_{i}^{2}$ is a bias of the *i*-th neuron of the first hidden layer, ${\omega }_{i,j}^{2}$ is a weight between the *i*-th neuron of the first hidden layer and the *j*-th neuron of the second hidden layer, ${b}_{j}^{3}$ is a bias of the *j*-th neuron of the second hidden layer, ${\omega }_{i}^{3}$ is a weight between the *j*-th neuron of the second hidden layer and the output layer, ${b}^{4}$ is a bias of the output layer.

In practice, the ANN compensator only performs forward propagation, and weights and biases are passed in real-time from the ANN identifier with the same network structure. These parameters are learned online by the backpropagation of the ANN identifier. To promote learning a precise inverse model of the controlled system, the Euclidean distance between the actual control law ${u}_{t}$ and the predicted control law ${\widehat{u}}_{t}$ is defined as the training loss function. Its expression is as follows

8 $$L=\frac{1}{2}{\left({u}_{t}-{\widehat{u}}_{t}\right)}^{2}$$

In the backpropagation process, the weight $\omega$ and bias $b$ of the ANN identifier are updated as follows

9 $$\omega \left(k+1\right)=\omega \left(k\right)-\eta \frac{\partial L}{\partial \omega }+\gamma \left[w\left(k\right)-w\left(k-1\right)\right]$$

10 $$b\left(k+1\right)=b\left(k\right)-\eta \frac{\partial L}{\partial b}+\gamma \left[b\left(k\right)-b\left(k-1\right)\right]$$

where $\eta$ is the learning rate, and $\gamma$ is the momentum factor that is introduced to speed up the network convergence and prevent the network from falling into a local optimum during the updating process.

After the ANN identifier learns the reverse model of the DC servo motor online using the above backpropagation process, the learned parameters are passed in real-time to the ANN compensator to generate the initial compensation control law ${u}_{n}$. Furthermore, to address the compensated uncertainty caused by the undertrained ANN, this study designs the FIS decider to adaptively generate the adjustment coefficient *c* for the ANN compensator. Its detailed principle is elaborated in the following section.

Finally, the algorithm of the FIS-enabled neural network feedforward compensation method is summarized in Table 1.

**Table 1:**
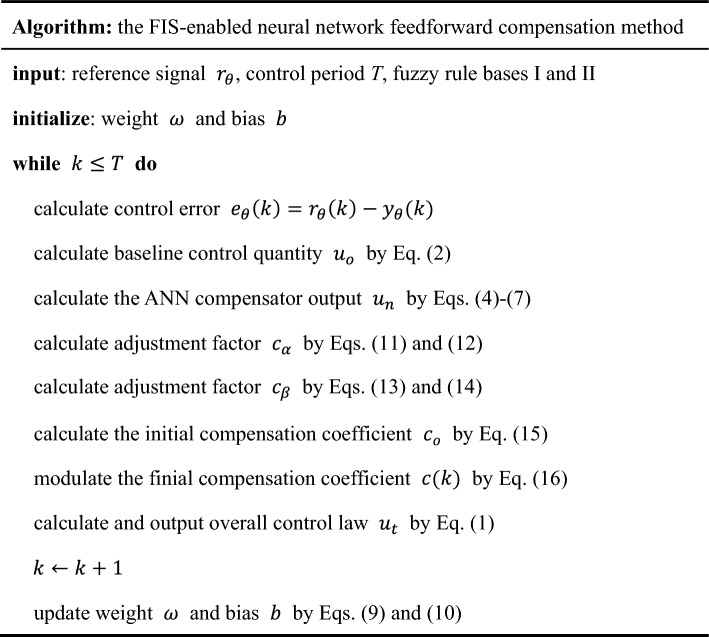
The algorithm of the proposed control method.

## FIS decider design

**Fig. 3:**
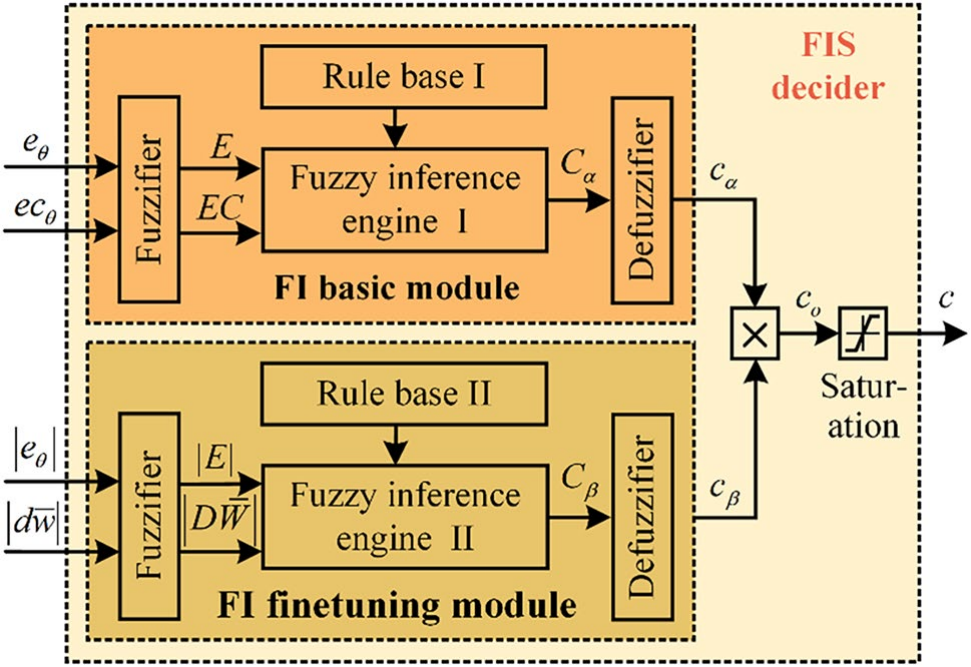
The overall structure of the designed FIS decider.

The designed FIS decider consists of the FI basic module and the FI finetuning module, and its structure is illustrated in Fig. 3. The FI basic module is firstly designed to weaken the uncertainty impact caused by the undertrained ANN to the control system. It deduces the coarse adjustment coefficient ${c}_{\alpha }$ for the compensated output of the ANN compensator according to the control error ${e}_{\theta }$ and error change ${ec}_{\theta }$ of the control system.

Since the scarcity of training samples at the leap moment of the tracking signal and the random initialization of the network parameters, the weight change of the ANN identifier causes the control system to destabilize to a certain extent. Correspondingly, the FI finetuning module is designed to further generate the adjustment coefficient ${c}_{\beta }$ according to the control error ${e}_{\theta }$ of the control system and the weight change ${dw}_{\theta }$ of the ANN identifier. Finally, under the joint action of the two modules, the control uncertainty of the ANN compensator is adjusted.

### FI basic module

The control error $e$ and its change $ec$ directly reflect the control performance of the DC servo motor system. To facilitate the stability of the control system, they are selected as the input variables of the FI basic module. Compared to the conventional Mamdani fuzzy model, a T-S-type FI with a simple structure has the advantage of clear reasoning and fast operation^40^. Therefore, the T-S-type FI is introduced into the designed FIS decider to suppress the control uncertainty of the ANN compensator in this study.

To balance the rule knowledge compatibility and the inference sensitivity, the input variables *e* and *ec* are divided into seven fuzzy sets: {Negative Big ($NB$), Negative Medium ($NM$), Negative Small ($NS$), Zero ($ZE$), Positive Small ($PS$), Positive Medium ($PM$), Positive Big ($PB$)}. The output variable is divided into five fuzzy sets: {Zero ($ZE$), Medium Small ($MS$), Medium ($M$), Medium Big ($MB$), Big Big ($BB$)}. To accelerate inference calculation, the output variable adopts 0-order T-S-type FI and is represented by five constant values. Concretely, the universe of input variables *e* and *ec* are [− 3, 3]. The universe of the fuzzy output variable ${C}_{\alpha }$ is [0,1], and the values corresponding to the five output fuzzy sets are shown in Table 2.

**Table 2:** The fuzzy set division of the output variable of the FI basic module.

Output	${C}_{\alpha 1}$	${C}_{\alpha 2}$	${C}_{\alpha 3}$	${C}_{\alpha 4}$	${C}_{\alpha 5}$
Fuzzy set	*ZE*	*MS*	*M*	*MB*	*BB*
Value	0	0.25	0.5	0.75	1

**Fig. 4:**
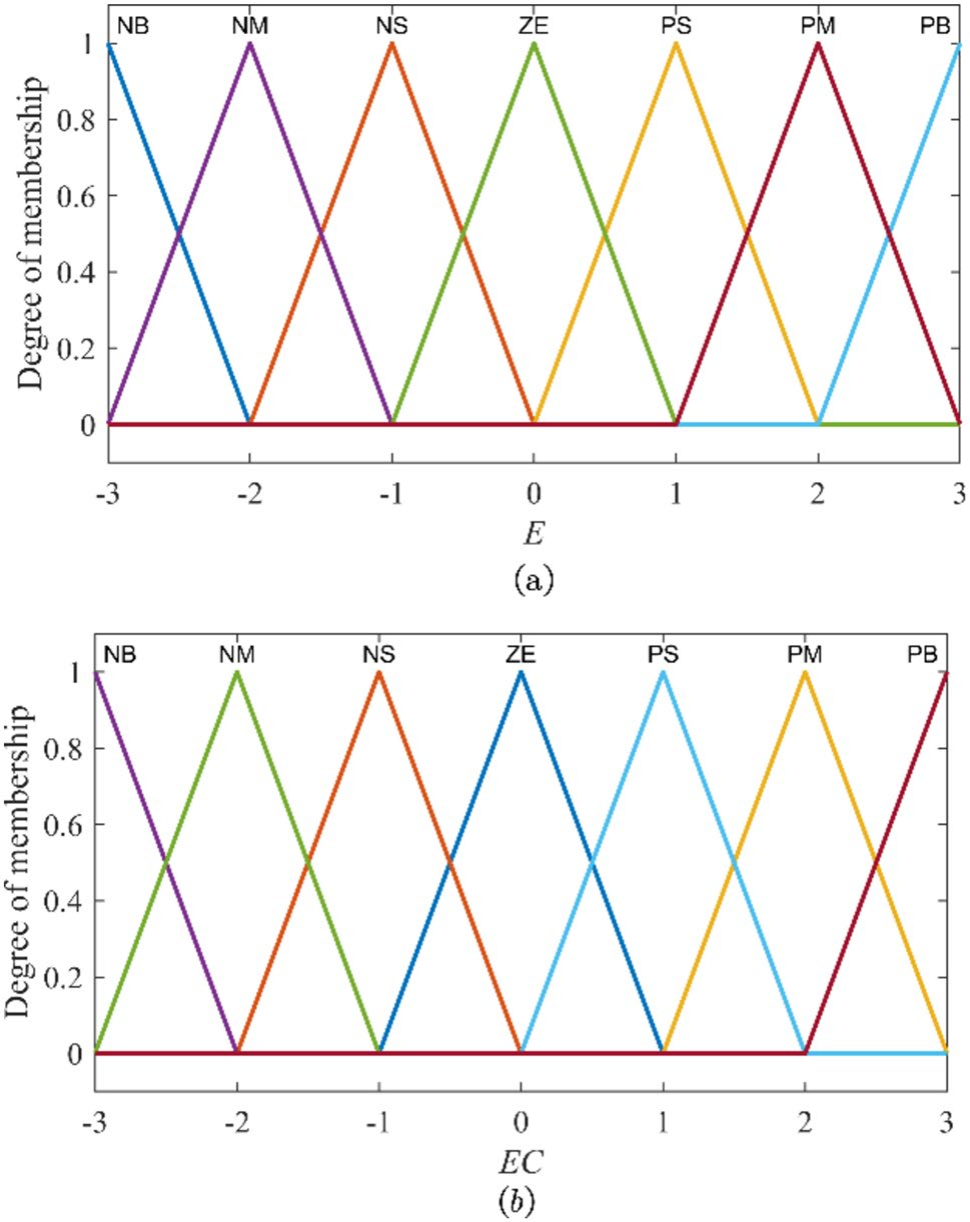
Membership functions in the FI basic module. (**a**) Membership function of fuzzy set *E*. (**b**) Membership function of fuzzy set *EC.*

The triangular membership function is frequently employed in the FIS due to its simple structure and ease of calculation^41^. Therefore, to enhance the FI operation speed, all seven fuzzy sets of the input variables $e$ and $ec$ adopt triangular functions as membership functions, as shown in Fig. 4.

For the FI basic module, the rule base I is designed based on the following principles:

At the initial moment of the control period and the leap moment of the tracking signal, the ANN identifier lacks sufficient training samples and can't learn the reverse model of the DC servo motor accurately. It leads the ANN compensator to hardly provide an efficient compensation quantity. The control error $e$ and its change $ec$ are large, resulting in a large uncertainty to the control system. Therefore, at this moment, the FI basic module is required to severely weaken the compensation output of the ANN compensator.

With the increase of online training samples, the ANN identifier learns the reverse model of the DC servo motor more accurately. The ANN compensator can provide more and more precise compensation quantity for the control system. At this stage, the control error $e$ and its change $ec$ gradually dwindle, thus the FI basic module needs to enhance the effect of the ANN compensator until the stability of the control system.

According to the above reasoning principles, the fuzzy rule base of the FI basic module is given in detail in Table 3.

**Table 3:** The fuzzy rule base I of the FI basic module.

		$$E$$						
		$NB$	$NM$	$NS$	$ZE$	$PS$	$PM$	$PB$
$EC$	$NB$	$ZE$	$ZE$	$MS$	$MS$	$MS$	$ZE$	$ZE$
	$NM$	$ZE$	$MS$	$M$	$M$	$M$	$MS$	$ZE$
	$NS$	$ZE$	$M$	$M$	$MB$	$M$	$M$	$ZE$
	$ZE$	$ZE$	$M$	$MB$	$BB$	$MB$	$M$	$ZE$
	$PS$	$ZE$	$M$	$M$	$MB$	$M$	$M$	$ZE$
	$PM$	$ZE$	$MS$	$M$	$M$	$M$	$MS$	$ZE$
	$PB$	$ZE$	$ZE$	$MS$	$MS$	$MS$	$ZE$	$ZE$

As the T-S-type FIS, the FI basic module uses the weighted sum method for defuzzification of the output variable. When the input variables $e$ and $ec$ activate *R* rules, the defuzzification of the output variable ${c}_{\alpha }$ is expressed as follows

11 $${c}_{\alpha }=\sum_{i=1}^{R}{\lambda }_{i}{C}_{\alpha i}$$

12 $${\lambda }_{i}={E}_{i}\left(e\right) {EC}_{i}\left(ec\right)$$

where ${\lambda }_{i}$ is the weight of the *i*-th rule. Besides, ${E}_{i}\left(e\right)$ and ${EC}_{i}\left(ec\right)$ are the membership degrees of the input quantities $e$ and $ec$ belonging to the fuzzy sets ${E}_{i}$ and ${EC}_{i}$.

Since control error $e$ and its change $ec$ reflect the approximating level of the inverse model by the ANN identifier passively, the FI basic module mainly adjusts the effect of the ANN compensator on the control system roughly.

### FI finetuning module

To further adjust the compensation quantity of the ANN compensator as precisely as possible, the FI finetuning module is designed in the FIS decider. Considering the weight change of the ANN directly impacts the output of the learned inverse model, therefore, the change of the weights between the last hidden layer and the output layer is introduced in the FI finetuning module. In addition, control error is used as a supplement input. Specifically, the FI finetuning module takes the absolute value of control error and weight change as input variables.

Similar to the FI basic module, to balance the rule knowledge compatibility and the inference sensitivity, the fuzzy input variables $\left|e\right|$ and $\left|d\overline{\omega }\right|$ are also divided into seven fuzzy sets: {Small Small ($SS$), Small Medium ($SM$), Small Big ($SB$), Medium ($M$), Big Small ($BS$), Big Medium ($BM$), Big Big ($BB$)}. To adjust the compensation quantity of the ANN compensator more precisely, the output variable is divided into seven fuzzy sets: {Small Small ($SS$), Small Medium ($SM$), Small Big ($SB$), Medium ($M$), Big Small ($BS$), Big Medium ($BM$), Big Big ($BB$)}. To accelerate inference calculation, the output variable still adopts 0-order T-S-type FI and is represented by seven constant values. Concretely, the universe of input variables $\left|e\right|$ and $\left|d\overline{\omega }\right|$ are [0, 3]. The universe of the fuzzy output variable ${C}_{\beta }$ is [0,1], the values corresponding to the seven output fuzzy sets are shown in Table 4.

**Table 4:** The fuzzy set division of the output variable of the FI finetuning module.

Output	${C}_{\beta 1}$	${C}_{\beta 2}$	${C}_{\beta 3}$	${C}_{\beta 4}$	${C}_{\beta 5}$	${C}_{\beta 6}$	${C}_{\beta 7}$
Fuzzy set	$SS$	$SM$	$SB$	$M$	$BS$	$BM$	$BB$
Value	0	0.5	0.6	0.7	0.8	0.9	1

**Fig. 5:**
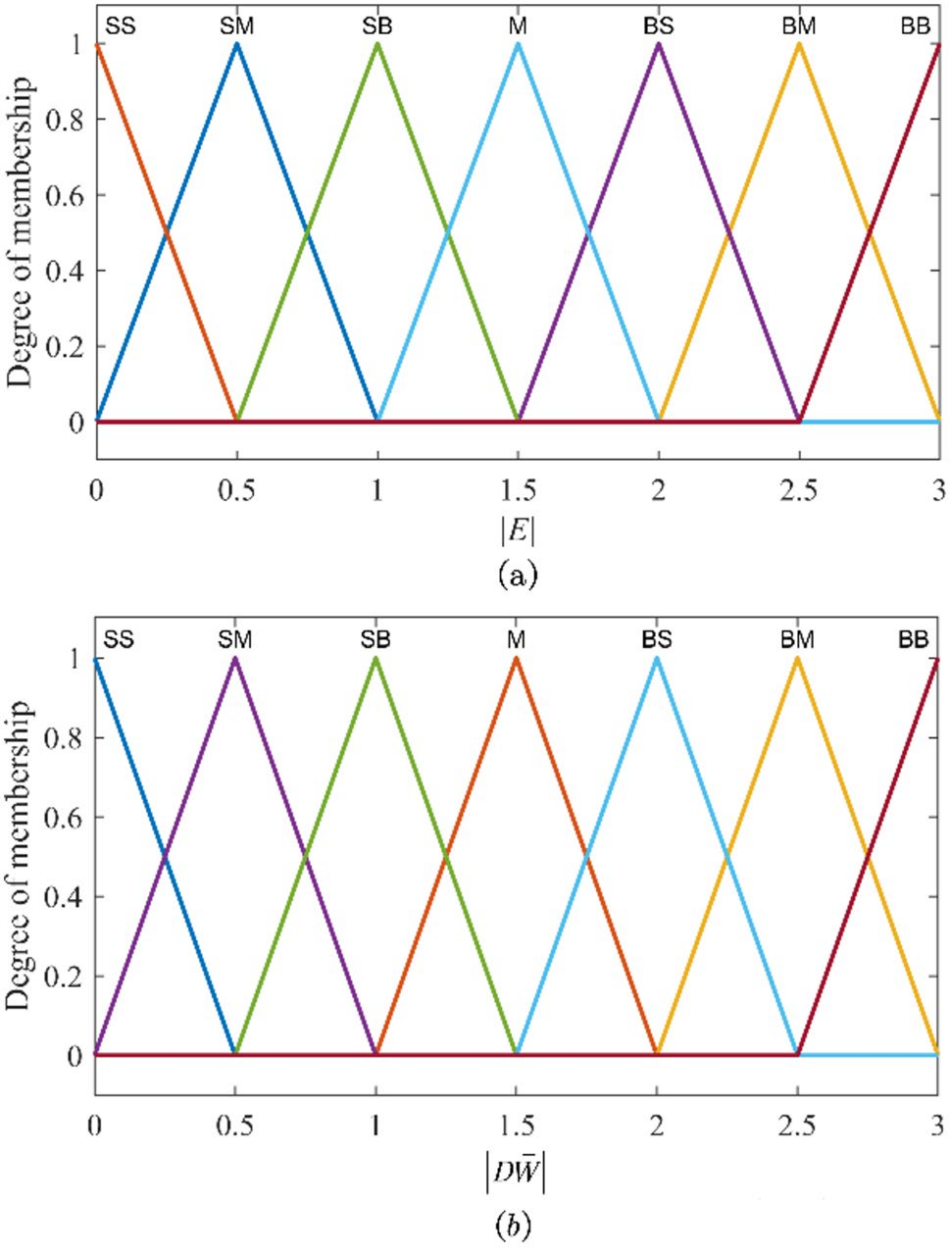
Membership functions in the FI finetuning module. (**a**) Membership function of fuzzy set $\left| E \right|$. (**b**) Membership function of fuzzy set $\left| {D\overline{W}} \right|$.

Similarly, to enhance the FI operation speed, all seven fuzzy sets of the input variables $\left|e\right|$ and $\left|d\overline{\omega }\right|$ still adopt triangular functions as membership functions, as shown in Fig. 5.

For the FI finetuning module, the rule base II is designed based on the following principles:

On the one hand, when the state of $\left|e\right|$ is the same as the state of $\left|d\overline{\omega }\right|$, that is, when $\left|e\right|$ is big, $\left|d\overline{\omega }\right|$ is also big, or when $\left|e\right|$ is small, $\left|d\overline{\omega }\right|$ is also small, the state change of the control system is synchronized with the state change of the ANN identifier. The FI basic module can efficiently adjust the compensation output, no additional adjustments are required from the FI finetuning module. At this stage, the reasoning output of the FI finetuning module is close to 1.

On the other hand, when the state of |e| and the state of $\left|d\overline{\omega }\right|$ are inconsistent, first, when $\left|e\right|$ is relatively big, but $\left|d\overline{\omega }\right|$ is relatively small, the training of the ANN is getting better and will converge soon, only minor suppression is required. On the contrary, when $\left|e\right|$ is small, but $\left|d\overline{\omega }\right|$ is big, the ANN is poorly trained, resulting in uncertainty in the output of the ANN compensator. Thus, it is necessary to further suppress the compensation output appropriately.

By leveraging the above reasoning principles, a total of forty-nine fuzzy rules can be obtained based on the input variables $\left|e\right|$ and $\left|d\overline{\omega }\right|$. Concretely, the fuzzy rule base II of the FI finetuning module is described in Table 5.

**Table 5:** The fuzzy rule base II of the FI finetuning module.

		$$\left|E\right|$$						
		$SS$	$SM$	$SB$	$M$	$BS$	$BM$	$BB$
$\left|D\overline{W }\right|$	$SS$	$BB$	$BB$	$BB$	$BB$	$BB$	$BB$	$BB$
	$SM$	$BM$	$BB$	$BB$	$BB$	$BB$	$BB$	$BB$
	$SB$	$BS$	$BM$	$BB$	$BB$	$BB$	$BB$	$BB$
	$M$	$M$	$BS$	$BM$	$BB$	$BB$	$BB$	$BB$
	$BS$	$SB$	$M$	$BS$	$BM$	$BB$	$BB$	$BB$
	$BM$	$SM$	$SB$	$M$	$BS$	$BM$	$BB$	$BB$
	$BB$	$SS$	$SM$	$SB$	$M$	$BS$	$BM$	$BB$

Similar to the FI basic module, the weighted sum method is applied for defuzzification of the output variable in the FI basic module uses. When the input variables $\left|e\right|$ and $\left|d\overline{\omega }\right|$ activate *R* rules, the defuzzification of the output variable ${c}_{\beta }$ is expressed as follows

13 $${c}_{\beta }=\sum_{i=1}^{R}{\varphi }_{i}{C}_{\beta i}$$

14 $${\varphi }_{i}={\left|E\right|}_{i}\left(\left|e\right|\right) {\left|D\overline{W }\right|}_{i}\left(\left|d\overline{\omega }\right|\right)$$

where ${\varphi }_{i}$ is the weight of the *i*-th rule. ${\left|E\right|}_{i}\left(\left|e\right|\right)$ and ${\left|D\overline{W }\right|}_{i}\left(\left|d\overline{\omega }\right|\right)$ are the membership degrees of the input quantities $\left|e\right|$ and $\left|d\overline{\omega }\right|$ belonging to the fuzzy sets ${\left|E\right|}_{i}$ and ${\left|D\overline{W }\right|}_{i}$.

Particularly, the reasoning output ${c}_{\beta }$ can essentially be considered an adaptive gain for the reasoning output ${c}_{\alpha }$ of the FI basic module. Therefore, after inferencing the adjustment factors ${c}_{\alpha }$ and ${c}_{\beta }$, the initial compensation coefficient ${c}_{o}$ is obtained as follows

15 $${c}_{o}={{c}_{\alpha }c}_{\beta }$$

Furthermore, to prevent violent jittering of the inference output, the initial compensation coefficient ${c}_{o}$ is followed by the saturation operation. Its calculation expression is as follows

16 $$c(k)=\left\{\begin{array}{c}{ c}_{o}\left(k\right)-\xi , { c}_{o}\left(k\right)-{c}_{o}\left(k-1\right)<-\xi \\ {c}_{o}\left(k\right), {|c}_{o}\left(k\right)-{c}_{o}\left(k-1\right)|\le \xi \\ {c}_{o}\left(k\right)+\xi , { c}_{o}\left(k\right)-{c}_{o}\left(k-1\right)>\xi \end{array}\right.$$

where $\xi$ represents the saturation coefficient of the change ratio of the adjustment factor $c(k)$.

After the saturation operation, the final adjustment factor $c(k)$ is used to adaptively adjust the compensation quantity of the ANN-based feedforward compensator. It is applied to improve the compensation precision in the position leap control of the DC servo motor and guarantee the transient stability of the control system.

## Experimental verification

### Experimental platform

**Fig. 6:**
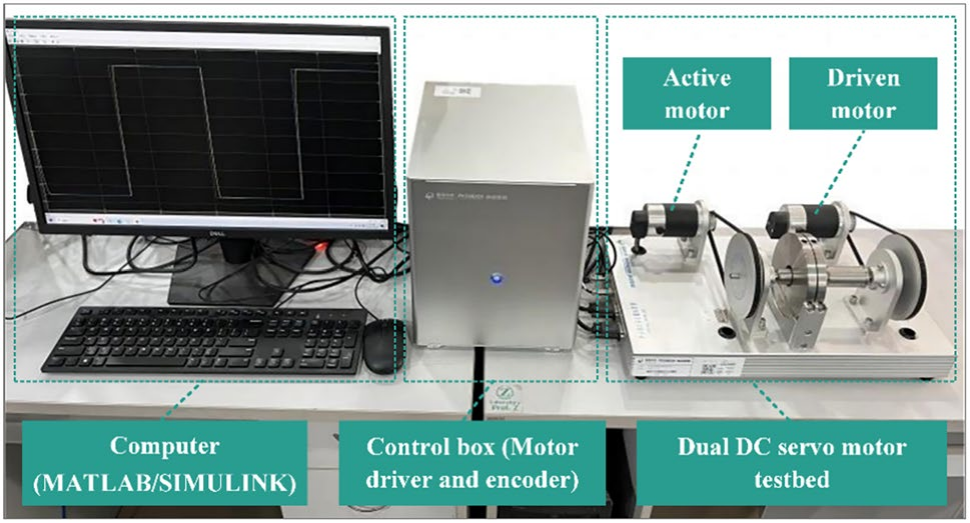
Experimental platform of the dual DC servo motor testbed.

To validate the effectiveness of the FIS-enabled ANN feedforward compensation method in real-time control, experimental research is conducted on the hardware-in-loop platform as shown in Fig. 6. It consists of a dual DC servo motor testbed, an integrated control box, and a computer. The motor testbed comprised an active motor and a driven motor. The control box includes a motor driver and a motor encoder. The motion control card is embedded in the computer through a PCI slot. In addition, the tracking controls of the step signal and square signal are carried out using the MATLAB/SIMULINK real-time workshop on the computer. Particularly, the DC servo motor used in the testbed is the T54H019 motor produced by Mingyago company, and its main parameters are shown in Table 6.

**Table 6:** Main parameters of the DC servo motor used in the testbed.

Item	Value	Unit
Armature inductance	0.0023	$\text{H}$
Armature resistance	3.44	$\Omega$
Torque constant	0.064	$\text{Nm}/\text{A}$
Rotational inertia	0.0000256	$\text{kg }{\text{m}}^{2}$
Back EMF coefficient	0.0068	$\text{V}/\text{r }{\text{min}}^{-1}$

In the control experiment, the motion control card controlled by MATLAB/SIMULINK real-time workshop sends an analog voltage to the motor driver within the control box. The driver converts the analog voltage into the armature current of the motor, generating torque and controlling the rotation of the motor.

Additionally, in the process of motor movement, the encoder collects the real-time position of the motor and feeds it back to the motor driver. After signal processing, the motor driver transmits the motor position in the form of pulses to the motion control card. The computer displays the motor position in real-time through the MATLAB/SIMULINK real-time workshop. According to the difference between the actual position and the expected position, the proposed method run on the MATLAB/SIMULINK real-time workshop calculates the corresponding control quantity and performs real-time control of the DC servo motor.

### Position tracking results

#### Step signal tracking performance

In this experiment, the PID parameters of the DC servo motor testbed are first adjusted to the optimal value as best as possible by using the trial-and-error method. Concretely, the PID parameters (${k}_{p}$, ${k}_{i}$, and ${k}_{d}$) of the motor position loop are set to 90, 0, and 20, and the PID parameters (${k}_{p}$, ${k}_{i}$, and ${k}_{d}$) of the motor speed loop are set to 8, 3, and 1.5, respectively. In particular, the PID parameters of the motor current loop can’t be turned by MTALAB, thus they are selected as the default value inside the motor driver. Note that all the control methods adopt the same PID parameters in the step signal tracking experiments to make the comparison fairer.

**Fig. 7:**
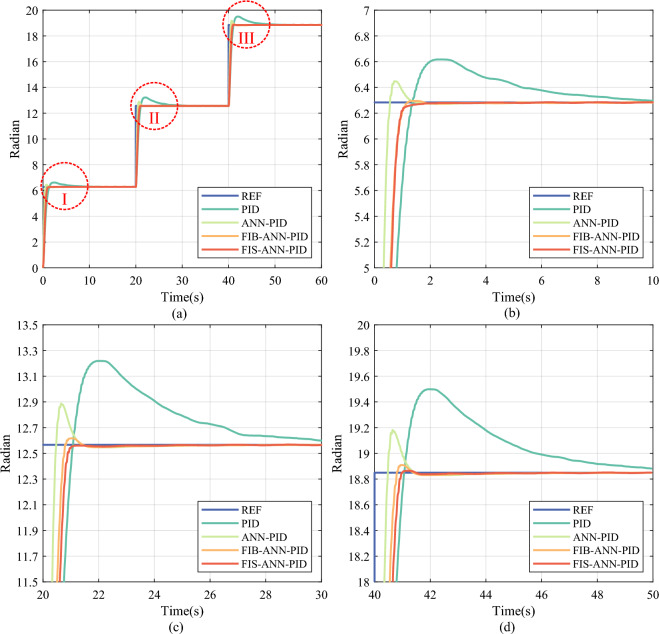
Experimental tracking results of step signal under different control methods. (**a**) Overall tracking result. (**b**) Partial enlarged detail I. (**c**) Partial enlarged detail II. (**d**) Partial enlarged detail III.

In addition, the learning rate *η* and the momentum factor $\gamma$ of ANN are set to $\eta =0.011$ and $\gamma =0.20$, respectively. In the FI basic module, the quantization factors of fuzzy variables *E* and *EC* are set to $5.0\times {10}^{-3}$ and $5.0\times {10}^{-5}$, respectively. In the FI finetuning module, the quantization factors of fuzzy variables *E* and *DW* are set to $5.0\times {10}^{-3}$ and $1.0\times {10}^{-5}$, respectively. The saturation coefficient $\xi$ of the change ratio of the adjustment factor is set to 0.005. Correspondingly, step signal tracking results using different control methods are depicted in Fig. 7, where ANN-PID represents the method consisting of the PID controller, the ANN identifier, and the ANN compensator. Besides, FIB-ANN-PID represents the method that adds the FI basic module based on ANN-PID. FIS-ANN-PID represents the proposed control method, that is, the method adds the FI finetuning module based on FIB-ANN-PID.

It can be seen from Fig. 7 that, in the step signal position tracking of the DC servo motor, the ANN-PID method lowers the overshoot and the setting time compared with the traditional PID method. Furthermore, the FIB-ANN-PID method and the FIS-ANN-PID method can improve the transient performance of the motor position control system compared to the ANN-PID method. Particularly, after introducing the FI finetuning module, the FIS-ANN-PID method realizes the small overshoot and the short settling time. Concretely, the quantitative comparison results of tracking step signals under different methods are summarized in Table 7, where SSME represents the steady-state mean error.

**Table 7:** Experimental comparison results of tracking step signals.

Position	Control method	Overshoot (Rad)	Settling time (s)	SSME (Rad)
I	PID	0.3336	5.1812	0.0213
	ANN-PID	0.1649	0.9185	0.0044
	FIB-ANN-PID	0.0122	0.9276	0.0034
	**FIS-ANN-PID**	**0.0000**	**0.9391**	**0.0031**
II	PID	0.6523	6.6243	0.0211
	ANN-PID	0.3211	1.0080	0.0053
	FIB-ANN-PID	0.0502	0.7264	0.0051
	**FIS-ANN-PID**	**0.0000**	**0.8601**	**0.0033**
III	PID	0.6484	6.3812	0.0240
	ANN-PID	0.3325	1.0236	0.0051
	FIB-ANN-PID	0.0593	0.7327	0.0052
	**FIS-ANN-PID**	**0.0149**	**0.8790**	**0.0033**

Significant values are given in bold.

As shown in Table 7, the ANN-PID method lowers SSME by 79.13%, 74.90%, and 78.69% compared to the PID method at the leap positions I, II, and III, respectively. It indicates that the ANN compensator can provide high-precision compensation for the position control of the DC servo motor when the control system into a steady state, benefitting from the ANN identifier can accurately learn the inverse model of the motor testbed online. When the ANN identifier can’t be fully trained by the insufficient samples at the position leap moment, the output of the ANN compensator has a large uncertainty, thereby the ANN-PID method still has a big overshot. After introducing the single FI basic module, the FIB-ANN-PID method lowers the overshoot by 96.35%, 92.30%, and 90.86% compared with the PID method at the leap positions I, II, and III, respectively. The results show that the FI module can suppress the compensation uncertainty of the ANN compensator caused by the undertrained ANN identifier.

**Fig. 8:**
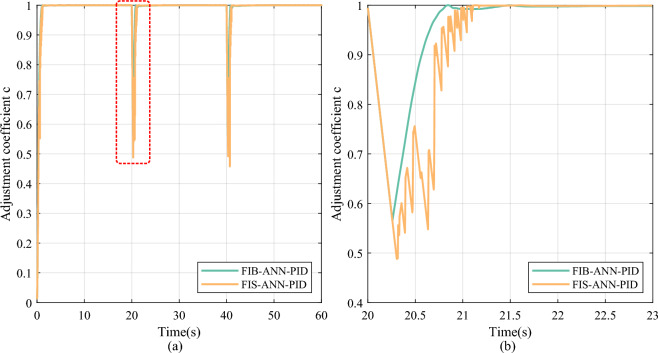
The change curve of the adjustment coefficient in the step signal tracking experiment. (**a**) Overall reasoning result. (**b**) Partial enlarged detail.

**Fig. 9:**
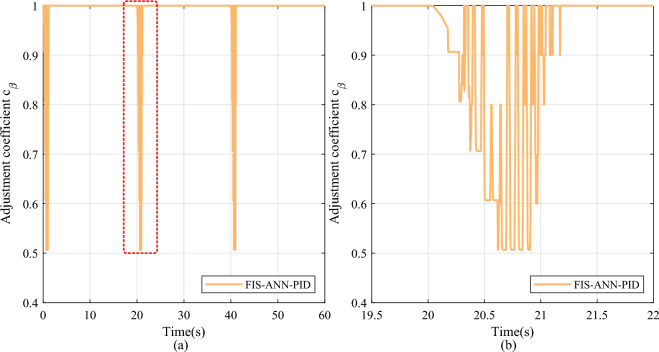
The change curve of the finetuning coefficient in the step signal tracking experiment. (**a**) Overall reasoning result. (**b**) Partial enlarged detail.

Furthermore, after adding the FI finetuning module, the FIS-ANN-PID method lowers the overshoot by 100% with little sacrifice in the settling time compared to the FIB-ANN-PID method at all leap positions. As shown in Figs. 8 and 9, the adjustment coefficient obtained by the combined action of the FI basic and finetuning module is more refined than that obtained by the single FI basic module at the position leap moment. It is because the FIS consisting of the FI basic and finetuning module considers not only the internal error and its change in the control system but also the change in the learned weights of the ANN identifier.

Consequently, the FIS-enabled ANN feedforward compensation method (i.e., FIS-ANN-PID) can not only enhance the dynamic quality but also improve the steady-state performance in the step signal tracking experiment of the DC servo motor.

#### Square signal tracking performance

In the square signal tracking experiment, the PID parameters of the DC servo motor testbed are also adjusted to the optimal value as best as possible by using the trial-and-error method. Concretely, the PID parameters (${k}_{p}$, ${k}_{i}$, and ${k}_{d}$) of the motor position loop are set to 90, 0, and 20, and the PID parameters (${k}_{p}$, ${k}_{i}$, and ${k}_{d}$) of the motor speed loop are set to 12, 6, and 1.5, respectively. Similarly, the PID parameters of the motor current loop are also selected as the default value inside the motor driver considering they can’t be turned by MTALAB/SIMULINK. Note that all the control methods adopt the same PID parameters in the square signal tracking experiments to make the comparison fairer.

**Fig. 10:**
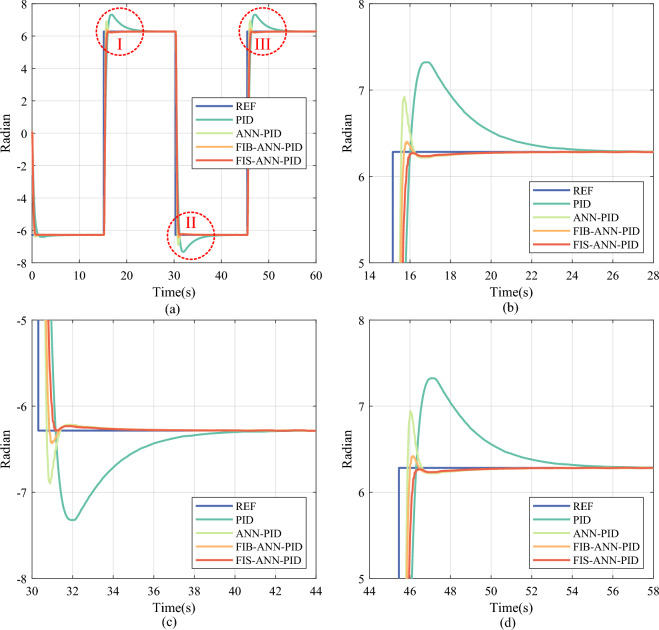
Experimental tracking results of square signals under different control methods. (**a**) Overall tracking result. (**b**) Partial enlarged detail I. (**c**) Partial enlarged detail II. (**d**) Partial enlarged detail III.

Additionally, the learning rate *η* and the momentum factor $\gamma$ of ANN are set to $\eta =0.011$ and $\gamma =0.12$, respectively. In the FI basic module, the quantization factors of fuzzy variables *E* and *EC* are set to $4.3\times {10}^{-3}$ and $6.7\times {10}^{-6}$, respectively. In the FI finetuning module, the quantization factors of fuzzy variables *E* and *DW* are set to $4.3\times {10}^{-3}$ and $1.0\times {10}^{-5}$, respectively. The saturation coefficient $\xi$ of the change ratio of the adjustment factor is set to 0.005. Correspondingly, square signal tracking results using different control methods are drawn in Fig. 10.

It can be found from Fig. 10 that, compared to the traditional PID method, the ANN-PID method lowers the overshoot and the setting time in the square signal position tracking of the DC servo motor. By adding the FI module based on the ANN-PID method, both the FIB-ANN-PID method and the FIS-ANN-PID method can achieve a small overshoot and a short settling time of the motor position control system. Moreover, after introducing the FI finetuning module, the FIS-ANN-PID method can improve the dynamic performance of motor position control compared to the FIB-ANN-PID method. Quantitatively, the comparison results of tracking square signals under different control methods are summarized in Table 8.

**Table 8:** Experimental comparison results of tracking square signal.

Position	Control method	Overshoot (Rad)	Settling time (s)	SSME (Rad)
I	PID	1.0380	4.7302	0.0264
	ANN-PID	0.6338	0.8214	0.0120
	FIB-ANN-PID	0.1211	0.4843	0.0122
	**FIS-ANN-PID**	**0.0000**	**0.6810**	**0.0096**
II	PID	1.0379	4.7201	0.0269
	ANN-PID	0.6146	0.8243	0.0121
	FIB-ANN-PID	0.1488	0.5013	0.0119
	**FIS-ANN-PID**	**0.0000**	**0.6790**	**0.0097**
III	PID	1.0397	4.7214	0.0281
	ANN-PID	0.6628	0.8521	0.0126
	FIB-ANN-PID	0.1396	0.4945	0.0121
	**FIS-ANN-PID**	**0.0000**	**0.6652**	**0.0104**

Significant values are given in bold.

As shown in Table 8, compared with the conventional PID method, the ANN-PID method lowers the overshoot by 38.94%, 40.78%, and 36.25%, reduces the settling time by 82.63%, 82.54%, 81.95%, and lowers the SSME by 54.37%, 55.13%, and 55.09% at positions I, II, and III, respectively. It testifies that the ANN compensator can implement a certain high-precision position compensation of the motor because the ANN identifier learns the inverse model of the motor testbed accurately online. But when the tracking signal suddenly changes, the training samples are significantly reduced, and the ANN identifier can’t learn the inverse model of the motor testbed accurately. At this moment, the compensation output of the ANN compensator has a large uncertainty, resulting in the ANN-PID method still having a big overshot.

After introducing the FI basic module, the FIB-ANN-PID method lowers the overshoot by 88.33%, 85.66%, and 86.58%, reduces the settling time by 89.76%, 89.38%, and 89.53%, and lowers the SSME by 53.64%, 55.52%, and 56.96% compared to the PID method at positions I, II, and III, respectively. It verifies that when the ANN identifier can’t learn the inverse model of the motor testbed effectively, the FI basic module can adjust the output uncertainty of the ANN compensator, thereby improving the dynamic performance of the motor control system.

**Fig. 11:**
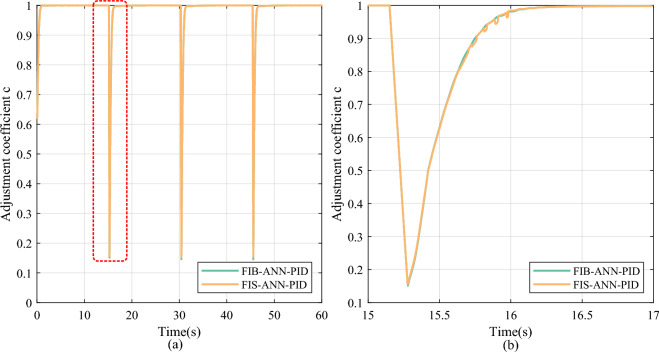
The change curve of the adjustment coefficient in the square signal tracking experiment. (**a**) Overall reasoning result. (**b**) Partial enlarged detail.

**Fig. 12:**
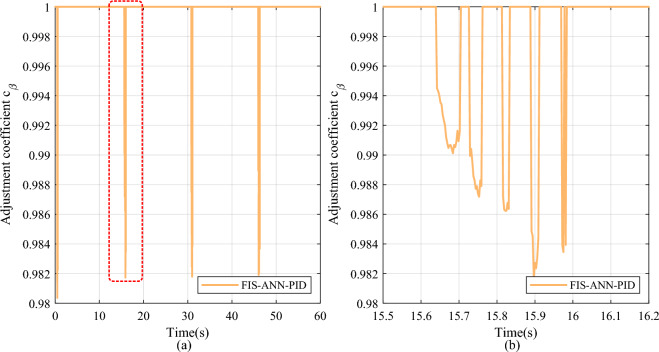
The change curve of the finetuning coefficient in the step signal tracking experiment. (**a**) Overall reasoning result. (**b**) Partial enlarged detail.

However, the FI basic module inferences the adjustment coefficient according to the error and its change in the whole control system, it only considers the internal state of the control system rather than the internal state of the ANN, especially the change in the learning weights. Therefore, as displayed in Figs. 11 and 12, compared with the single FI basic module designed in the FIB-ANN-PID method, the FIS decider consisting of the FI basic and finetuning modules can deduce the more imperceptible details.

Furthermore, by combining the FI basic and finetuning modules, the FIS-ANN-PID lowers the overshoot by 100.00%, 100.00%, and 100.00%, reduces the settling time by 85.60%, 85.61%, and 85.91%, and lowers the SSME by 63.56%, 63.90%, and 63.11% compared to the PID method at positions I, II, and III, respectively. It significantly reduces the overshoot and improves the steady-state accuracy without spending almost any extra settling time, compared to the ANN-PID method and the FIB-ANN-PID method. Ultimately, in the square signal tracking experiment of the DC servo motor, the FIS-ANN-PID method proposed in this study improves both the dynamic performance and the steady-state accuracy.

## Discussion

### Hyperparameter sensitivity

**Fig. 13:**
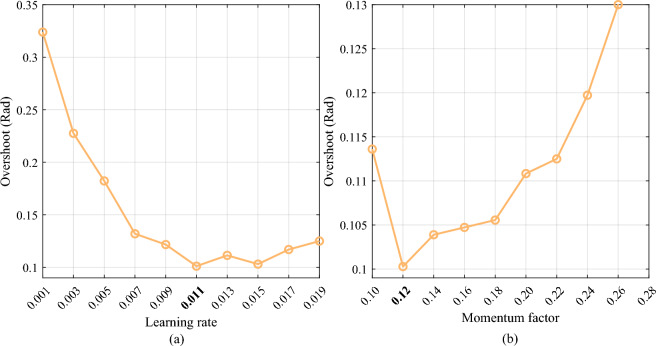
Sensitivity results of different learning rates and different momentum factors. (**a**) Sensitivity of different learning rates. (**b**) Sensitivity of different momentum factors.

As shown in Eqs. (9) and (10), the learning rate η and the momentum factor γ are key parameters to make the ANN identifier learn the reverse model of the DC servo motor accurately. Take the square signal tracking experiment as an example, the sensitivity results of different learning rates and different momentum factors are shown in Fig. 13.

It can be found from Fig. 13 that, when the learning rate is less than 0.011 in the square signal tracking experiment, the lower the learning rate, the larger the overshoot, and then the transient performance of the control system becomes poorer. When the learning is larger than 0.011, the overshoot increases with the increase in the learning rate. Therefore, the learning rate is determined as 0.011 in the tracking experiment. In addition, when the momentum factor is equal to 0.12, the overshoot of the control system is lowest, therefore, it is determined as the appropriate value in the tracking experiment.

### Anti-interference performance

**Fig. 14:**
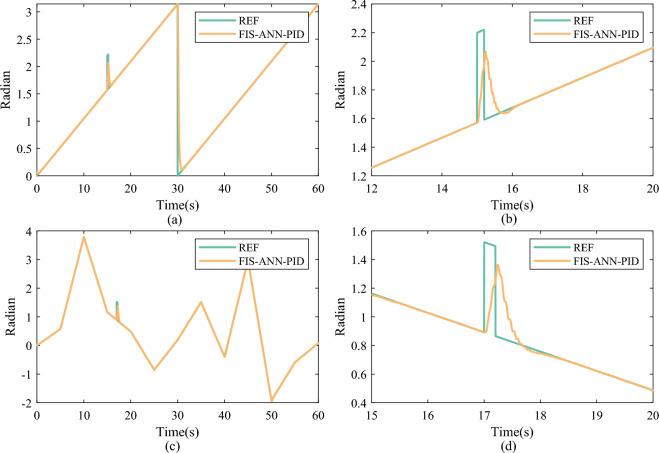
Anti-interference experiment results of under sawtooth and stochastic signals. (**a**) Overall tracking result under sawtooth signal. (**b**) Partial enlarged detail under sawtooth signal. (**c**) Overall tracking result under stochastic signal. (**d**) Partial enlarged detail under stochastic signal.

To evaluate the anti-interference performance of the proposed FIS-enabled ANN feedforward compensation control method, the disturbance experiments under tracking sawtooth and stochastic signals are carried out in the DC servo motor testbed. The experiment results are drawn in Fig. 14, where the two left subgraphs display the overall tracking results, and the two right subgraphs exhibit partial enlarged details.

As shown in Fig. 14a,b, the DC servo motor can quickly respond to the disturbing reference when tracking the sawtooth signal. Additionally, as shown in Fig. 14c,d, upon encountering a disturbance during the tracking experiment of the stochastic signal, the DC servo motor also promptly adjusts to the altered reference. Therefore, the proposed control method demonstrates excellent anti-interference performance benefitting from the quick inference of the FIS decider as well as accurate compensation of the ANN compensator in real-time.

## Conclusion

In this study, the FIS-enabled ANN feedforward compensation method is proposed to realize the high-performance position leap control of the DC servo motor. In the method, the ANN identifier is built to accurately learn the reverse model of the DC servo motor control system while the ANN compensator sharing the same network structure is designed to online provide high-precision feedforward compensation quantity. Furthermore, considering both the system tracking error and network modeling error, the FIS consisting of an FI basic module and an FI finetuning module is developed to adjust the ANN feedforward compensation quantity and prevent the uncertain disturbance of the undertrained ANN automatically.

Experimental results show that when tracking the step and square signals, the proposed method lowers the overshoot by an average of 98.50% and 100%, reduces the settling time by an average of 8.85% and 18.89%, and lowers the steady-state error by an average of 33.51% and 19.17%, compared with the ANN method under the same conditions, respectively. It demonstrates the proposed method not only enhances the dynamic performance but also improves the steady-state accuracy of the DC servo motor control system significantly. Therefore, the FIS-enabled ANN feedforward compensation method is feasible and effective. Particularly, it is suitable for time-varying control systems that cannot be accurately modeled and easy to perform tracking control of the signals with abrupt change characteristics.

For future works, on the one hand, to guarantee the high synchronization performance of two DC servo motors^42^, a fuzzy inference-based ANN feedforward compensation method via integrating adaptive backstepping control^43^ is worth being developed for dual DC servo motor systems. On the other hand, a variable universe fuzzy inference decided ANN feedforward compensation method is also worth further being investigated for the high-precision position control of DC servo motor systems.

## Data Availability

All data generated or analyzed during this study are included in this published article. And the data that support the findings of this study are available from the corresponding author on reasonable request.
